# Undocumented Americans Need Equitable Language in Worker Training

**DOI:** 10.1089/heq.2022.0006

**Published:** 2022-08-29

**Authors:** Eric Persaud

**Affiliations:** South Richmond Hill, New York, USA.

Dear Editor:

Undocumented immigrant American workers face barriers to adequate safety training and disparities in occupational health.^1^ The workplace plays a vital role in the lives of all Americans who perform the necessary work that keeps society functioning, including undocumented workers. It is important that workers receive training and education to perform their work safely and in a healthy manner.

Since the COVID-19 pandemic, there has been a shift from in-person training and education to virtual platforms due to the need to be socially distant, including occupational health and safety (OHS) training. There was a need to integrate COVID-19 and infectious disease control and prevention curricula into broader worker training programs. Some of those OHS trainings were conducted virtually, despite barriers that existed for many, including immigrants and persons of color, in technological access, comfort, and fluency.^2^

Undocumented workers, often immigrants and persons of color, filled many of the roles of essential workers, and were disproportionately employed to work in-person during the COVID-19 pandemic in unsafe working conditions and environments.^3^ It remains unclear the effectiveness of OHS training undocumented workers received in response to the COVID-19 pandemic and in some cases if any OHS training was even offered. Public health practitioners, researchers, and program planners need to further recognize how the COVID-19 pandemic has created changes in training and education for undocumented workers, who were already facing limitations in virtual and in-person services.

At a minimum, the language for OHS training needs to be appropriate for the audience and competently delivered by the instructor.^4^ Language, however, is not just the dialect, for example, English, Spanish, or Vietnamese, but also using the words in a context the trainees understand.^5^ For example, undocumented workers may fear reporting injuries on the jobsite and unsafe working conditions, despite their right to do so under federal law without employer retaliation. Simply stating a worker can report injuries may not be enough for undocumented workers. Clarifying worker rights against employer retaliation regardless of their documentation status is important and should be emphasized in OHS training programs.^1^ This is one of many such examples of how language of worker rights to safety and health needs to be equitable to undocumented workers.

There is a need to act toward ensuring that language equity is part of OHS training. Instructors need to engage those with lived experiences of undocumented status and advocate for such audiences to support review of curricula. The input of those with lived experiences can provide context and content an instructor may not have otherwise. Instructors should develop and deliver OHS training on a platform and in a language participants understand, provide translation of materials into languages that fit possible target audiences, and use terminology framed in a practical context applicable to the target audience. To do so would better prepare and protect all workers, including our undocumented workers who equally deserve and need safe working conditions and environments.

## Abbreviation Used

OHS: occupational health and safety
